# A Sensor-Based Classification for Neuromotor Robot-Assisted Rehabilitation

**DOI:** 10.3390/bioengineering12030287

**Published:** 2025-03-13

**Authors:** Calin Vaida, Gabriela Rus, Doina Pisla

**Affiliations:** 1 CESTER—Research Center for Industrial Robots Simulation and Testing, Technical University of Cluj-Napoca, 400114 Cluj-Napoca, Romania; calin.vaida@mep.utcluj.ro (C.V.);; 2 Technical Sciences Academy of Romania, B-dul Dacia, 26, 030167 Bucharest, Romania

**Keywords:** neuromotor robot-assisted rehabilitation, sensors, patient-centered rehabilitation, personalized medicine

## Abstract

Neurological diseases leading to motor deficits constitute significant challenges to healthcare systems. Despite technological advancements in data acquisition, sensor development, data processing, and virtual reality (VR), a suitable framework for patient-centered neuromotor robot-assisted rehabilitation using collective sensor information does not exist. An extensive literature review was achieved based on 124 scientific publications regarding different types of sensors and the usage of the bio-signals they measure for neuromotor robot-assisted rehabilitation. A comprehensive classification of sensors was proposed, distinguishing between specific and non-specific parameters. The classification criteria address essential factors such as the type of sensors, the data they measure, their usability, ergonomics, and their overall impact on personalized treatment. In addition, a framework designed to collect and utilize relevant data for the optimal rehabilitation process efficiently is proposed. The proposed classifications aim to identify a set of key variables that can be used as a building block for a dynamic framework tailored for personalized treatments, thereby enhancing the effectiveness of patient-centered procedures in rehabilitation.

## 1. Introduction

Neurological diseases are one of the main causes of neuromotor disabilities which require specific rehabilitation programs [1,2]. The scientific literature shows that the diseases benefiting the most from neuromotor rehabilitation can be categorized in three main groups: *Vascular group*—which are mainly circulatory system diseases (e.g., stroke, peripheral artery disease); *Extrapyramidal group*—composed of diseases related to the brain structures involved in regulation and coordination of movement (e.g., Parkinson, Dystonia); *Neuromuscular group*—includes those disorders who affect both the nervous system and the muscles (e.g., different form of sclerosis, Guillain–Barré syndrome) [3].

Considering that neurological disorders account for most long-term disabilities, an additional challenge is that neurological disorders are particularly common among the older population, a population whose number is steadily rising [4,5]. This particular aspect is very important considering that the World Health Organization (WHO) estimated the segment of the population composed of people around 80 years or over will be increased from 143 million in 2019 to 426 million in 2050 [4]. As part of ongoing efforts, various strategies have been developed to prevent neurological diseases and improve the lives of those already affected by disabilities [2,5,6]. Many studies have shown that factors associated with the standard of living are strongly correlated with the likelihood of developing neurological diseases [6,7,8,9]. Therefore, preventive strategies include public health campaigns, investments in healthcare infrastructure, and community programs aimed at raising awareness among the population about the associated risks [5].

**Figure 1:**
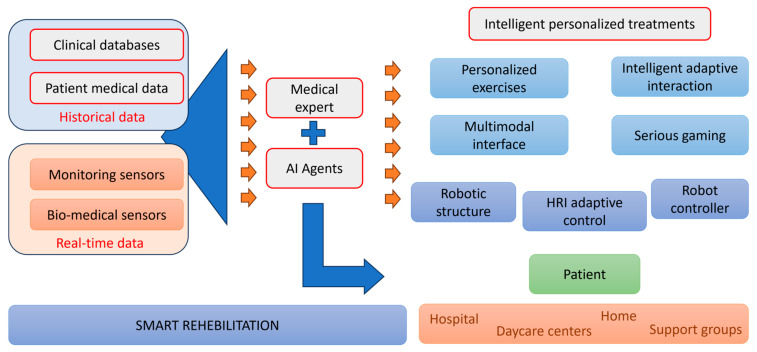
The paradigm of robotic-assisted rehabilitation.

Despite all these efforts, there is a large increase in the number of patients with different neuromuscular disorders which are pressuring the medical system up to a point where patients cannot benefit from proper care. At the level of the European Union a paradigm change has been suggested since 2014, when robotic assisted rehabilitation has been included in the strategic development agenda and refined in the Joint Strategic Research Innovation and Deployment Agenda (SRIDA) for the AI, Data, and Robotics Partnership [10]. These documents are clear demonstrators of the paradigm change in medical rehabilitation, introducing robotic devices as advanced therapeutic solutions as illustrated in Figure 1.

Based on the paradigm change (Figure 1), robotic-assisted rehabilitation can be seen as a technology enabler for real-time and historical data management allowing the collection of various patient parameters data using different types of sensors and storage of this information in large databases. The intelligent processing of these data supports the therapy transformation from “gold standards” toward personalized treatment programs [11,12,13,14].

Numerous studies have explored the use of wearable sensors, robotics, and virtual reality to collect data on specific rehabilitation parameters such as force, range of motion, and muscle activity [15,16,17,18,19,20,21,22]. Additionally, research has investigated the role of non-specific parameters, including temperature, oxygen saturation, and heart rate, in influencing rehabilitation outcomes [23,24,25]. However, to the authors’ knowledge, there is no comprehensive framework that integrates both specific and non-specific parameters into a unified classification system oriented towards patient conditions and comorbidities.

The main objective of this review is to organize information and data collection methods systematically. In doing so, the authors aim to conceptualize a novel framework for classifying and integrating sensors into personalized neuromotor robot-assisted rehabilitation. By effectively monitoring patients’ current conditions and progress, the proposed framework lays the groundwork for a new paradigm in rehabilitation. This framework, enabled by advanced sensor technologies (Figure 1), not only personalizes treatment but also extends its reach beyond traditional environments (rehabilitation centers, hospitals) to include telerehabilitation at home or daycare centers. By utilizing real-time data from sensors, this approach ensures efficient and personalized care delivery to a broader population, including tele-rehabilitation. The proposed framework introduces a classification method that categorizes parameters into specific (e.g., force, muscle contraction) and non-specific (e.g., temperature, oxygen saturation) to better adapt rehabilitation strategies to individual patient profiles. The contributions of the paper with respect to the state of the art and the patient-centered approach to robotic-assisted rehabilitation are as follows:

(i) Analyzes the methods and reliability in collecting specific and non-specific parameters in rehabilitation treatment.
(ii) Classifies the sensors according to their types, the data they collect, their usability, and ergonomics.
(iii) Assesses the impact of comorbidities on treatment rehabilitation.
(iv) Evaluates the most efficient methods for data acquisition and utilization.

Following the Introduction, which highlights the role of sensors in personalized neuromotor robot-assisted rehabilitation, Section 2 classifies sensors based on their type, the data they measure, their usability, and ergonomics, focusing on their role in motor function assessment. Section 3 expands the classification by addressing sensors that measure non-specific physiological and metabolic parameters, which influence rehabilitation outcomes indirectly. Section 4 explores how comorbidities shape rehabilitation strategies, emphasizing sensor-driven personalization. Section 5 details real-time data acquisition and AI-driven processing for adaptive therapy. Section 6 integrates these findings into real-world applications, addressing challenges like interoperability and patient adherence, leading to Section 7, which summarizes key insights and outlines future research directions.

## 2. Methodology

### 2.1. Literature Review Methodology

**Figure 2:**
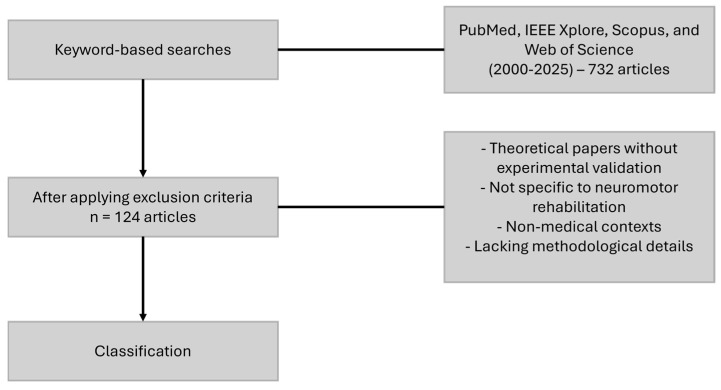
Article selection method.

This review followed a structured methodology, including systematic literature searches across multiple databases, predefined inclusion/exclusion criteria, and a classification-based analytical approach. This methodology can be seen in Figure 2. Scientific publications were identified through keyword-based searches in PubMed, IEEE Xplore, Scopus, and Web of Science, covering the period 2015–2025. An initial dataset of 732 articles was identified, which was systematically filtered using predefined exclusion criteria, resulting in a final selection of 124 articles for analysis.

The search terms include combinations of the following:

- **Sensor-based rehabilitation** (“rehabilitation sensors“, “robot-assisted rehabilitation”, “sensor technology in rehabilitation”)
- **Wearable and non-wearable sensors** (“wearable rehabilitation devices”, “non-invasive sensors”, “implantable biosensors”)
- **Motion and force tracking** (“motion tracking sensors”, “force sensors in rehabilitation”, “gait analysis sensors”)
- **Bioelectrical and neurophysiological sensors** (“electromyography in rehabilitation”, “EEG for neurorehabilitation”)
- **Real-time data processing and AI-driven feedback** (“real-time rehabilitation feedback”, “AI in rehabilitation sensors”, “sensor fusion in neurorehabilitation”)
- **Comorbidity-driven sensor applications** (“sensors for metabolic disorders”, “neurological disease monitoring”, “rehabilitation in chronic pain conditions”)
- **Physiological and metabolic monitoring in rehabilitation** (“oxygen saturation in rehabilitation”, “cardiac monitoring for therapy adaptation”, “sweat analysis for patient assessment”)

A total of 102 articles were selected based on their relevance to the following key aspects:

**Sensor technology and classification**—Studies that described the type, function, and characteristics of the sensors used in neuromotor robot assisted rehabilitation.

**Sensor usability and ergonomics**—Research that examined ease of use, adaptability, and comfort for patients.

**Sensor performance metrics**—Papers that evaluated accuracy, response time, and reliability in real-world rehabilitation settings.

**Clinical integration of sensors**—Studies that demonstrated how specific sensors contribute to patient-centered rehabilitation approaches.

Studies that only focused on theoretical models without experimental validation were excluded, as were articles that did not specifically address neuromotor rehabilitation applications. Additionally, studies analyzing sensors in non-medical contexts or without a clear connection to neuromotor rehabilitation were not considered. Articles lacking sufficient details about the methodologies used were also excluded.

### 2.2. Analysis and Categorization Process

A qualitative thematic analysis was applied to identify common attributes across different sensor types. This process involved two main steps:

#### 2.2.1. Data Extraction and Organization

Each selected article was reviewed to extract key sensor-related information, including sensor type and measurement principle, the parameters it records (e.g., motion, force, bioelectrical activity) its usability, invasiveness, and cost considerations.

#### 2.2.2. Classification Development

**Figure 3:**
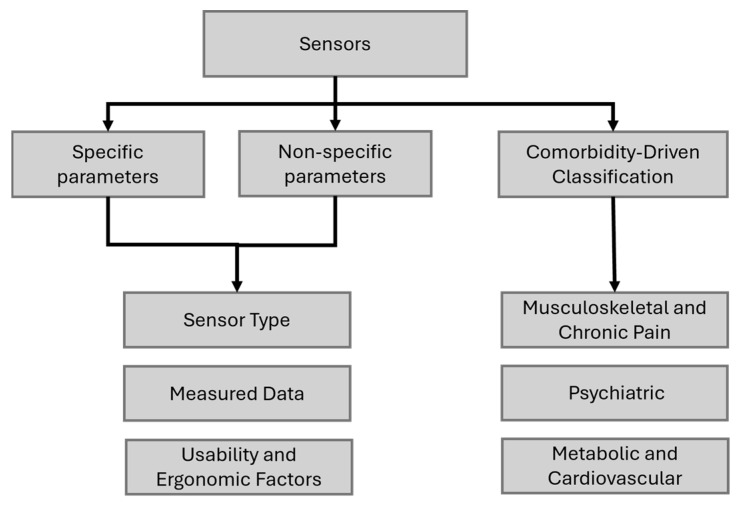
The proposed classification.

The primary classification categorizes sensors into three main groups: those measuring specific parameters, those measuring non-specific parameters, and sensors designed for treatment adjustments in patients with specific comorbidities. Furthermore, specific and non-specific parameters are classified based on sensor type, measured data, usability, and ergonomics, while the comorbidity-driven classification is divided into musculoskeletal and chronic pain, psychiatric, and metabolic and cardiovascular categories. The proposed classification can be seen in Figure 3.

**(a)**  **Specific Parameters**

These parameters define the type of data measured by the sensors and their functional role in rehabilitation, being directly related to rehabilitation measurements.

They are classified based on sensor type (motion sensors, force sensors, bioelectrical sensors, optical sensors, etc.), measured data (biomechanical tracking, electrophysiological signals, metabolic monitoring) and usability and ergonomic factors (wearability, complexity, cost-effectiveness).

**(b)**  **Non-Specific Parameters**

These parameters contribute indirectly to rehabilitation by providing additional physiological or metabolic insights. Often used for patient monitoring, health status assessment, and therapy adaptation. The classification structure for non-specific parameters follows the same hierarchical organization as that of specific parameters, ensuring consistency across sensor categorization.

**(c)**  **Comorbidity-Driven Classification**

This category addresses how sensor selection is influenced by pre-existing conditions affecting the rehabilitation process. Patients with neurological, musculoskeletal, metabolic, or cardiovascular comorbidities may require specific sensor adjustments for optimal therapy. Sensors are classified based on their ability to support personalized rehabilitation strategies for these conditions.

Due to the multifunctional nature of certain sensors, some devices were categorized under multiple classifications. Sensor overlap was deliberately maintained to reflect their dual-purpose applications in rehabilitation settings.

## 3. Classification of Sensors for Specific Motor Parameters in Rehabilitation: Type, Measured Data, Usability, and Ergonomics

Considering the critical role of sensors in personalizing treatment, it is essential to evaluate their contribution to robot-assisted rehabilitation. To properly understand these sensors’ potential advantages and disadvantages and guarantee their effective integration into patient-centered rehabilitation approaches, a thorough evaluation is required.

Thus, to provide a proper overview of their implication in the treatment, a classification based on the type of the sensor, on the measurements of the collected data, of the usability of the sensors, and on ergonomics, is provided.

### 3.1. Classification Based on Type

**Motion sensors**

➢ Inertial Measurement Units—in the context of rehabilitation, these sensors are used for motion tracking and analysis, functional movement assessment, and real-time feedback [26,27,28,29].
➢ Accelerometers are used to measure the acceleration of a moving body, detecting changes in velocity and direction. These types of sensors are used to track movement patterns, movement analysis, and balance assessment [30,31].
➢ Gyroscopes are used to measure orientation and angular velocity, providing information about the joint movements and changes in posture [32].
➢ Optical motion tracker systems are based on optical technology being used for real time gait measurement, estimation of upper and lower extremity kinematics or postural control analysis, and interaction with virtual reality environments for rehabilitation [33,34,35,36,37,38].

**Force Sensors**

➢ Pressure sensors are used to assess posture, hand joints stiffness, finger angle estimation, seating dynamics, and gait analysis [39,40,41].
➢ Load cell are employed in assessing muscle strength, monitoring weight-bearing exercises, and evaluating balance during standing or walking [42,43].

**Electromechanical sensors**

➢ Capacitive—due to their flexibility can be used in measuring the joint motions, monitoring higher pressures such as plantar pressure, monitoring the joints angles [44,45,46].
➢ Triboelectric—converting mechanical energy into electrical energy, these sensors can respond to external pressure stimuli, thus being suitable for applications which involve the measuring of the plantar pressure, pressing and stretching force of the hand or gait monitoring [47,48,49].
➢ Piezoresistive—these types of sensors are often encountered in applications where detecting subtle movements and pressures during rehabilitation exercises is crucial and can be used in applications such as measurement and analysis of the plantar pressure force, manipulator soft grabbing, and human movement monitoring or touch-based game systems for upper-limb rehabilitation [50,51,52].
➢ Piezoelectric are used for the assessment and motion identification of wrist joint rehabilitation training, muscle activity monitoring and real-time feedback from patients [53,54].

**Bio-electrical sensors**

➢ Electromyography (EMG) sensors are usually used to create a brain–computer interface (BCI) to detect electrical activity associated with muscle contraction, to regain the partial loss function of certain muscles or to control external devices, such as prosthesis [55,56,57,58].
➢ Electromyography (EEG) sensors: Similarly, electroencephalography (EEG) sensors are integral to BCI systems, facilitating direct communication between the brain and external devices. They offer various applications including neurofeedback training for cognitive rehabilitation, assistance in telerehabilitation, and interaction with gaming or virtual reality environments [59,60,61].

### 3.2. Classification Based on Measured Data

**Figure 4:**
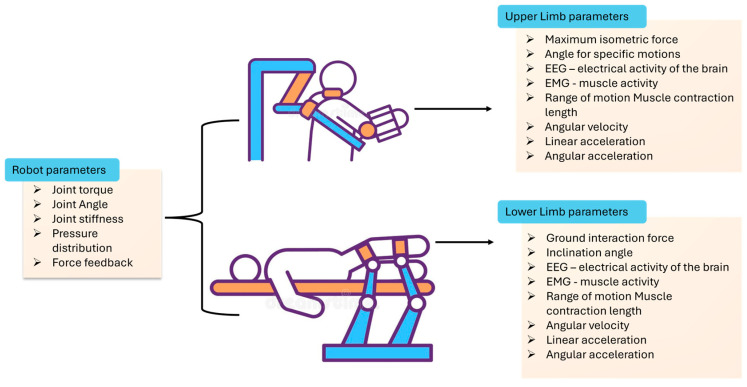
A schematic representation of the targeted parameters from both patient and robots used for neuromotor robot-assisted rehabilitation.

A classification of the data collected by the discussed sensors is presented in Table 1. Measured parameters are categorized based on their respective areas of utilization: parameters measured on the robotic structures used in rehabilitation, and parameters measured on both the upper and lower limbs during motion, as can be seen in Figure 4. This classification aims to offer an overview of the targeted signals by physicians in current procedures.

### 3.3. Classification Based on Usability and Ergonomics

A number of articles were analyzed for this classification, where the performance of the sensors has been clearly specified [18,50,69,70,71].

**High Usability**—characterized by intuitive interfaces and minimal setup requirements.

➢ *Sensors*: Inertial measurement unit (IMU)s, optical motion tracker systems, capacitive sensors, piezoelectric sensors, EMG sensors, EEG sensors.

**Ergonomically Designed**—These sensors include adjustable components, lightweight materials, and designs that promote comfort during prolonged use.

➢ *Sensors:* IMUs, optical motion tracker systems.

**High Accuracy**—These sensory devices feature high-precision components as well as advanced calibration mechanisms that guarantee reliable and accurate measurement outputs in different environments.

➢ *Sensors*: Optical sensors, force sensors, EMG sensors, EEG sensors.

**Real-time feedback**—They possess processing capacities of real time data which include analyzing and giving instant responses in physiotherapy sessions.

➢ *Sensors*: IMUs, optical sensors, force sensors, EMG sensors, EEG sensors, gyroscopes, accelerometers.

**Non-invasiveness**—designed to be non-invasive, meaning they can be applied or used on the surface of the body or objects without requiring insertion or penetration.

➢ *Sensors*: IMUs sensors, accelerometers, gyroscopes, optical motion tracker systems, pressure sensors, capacitive sensors, triboelectric sensors, piezoresistive sensors, piezoelectric sensors, EMG sensors (electromyography), EEG sensors (electroencephalography).

**Cost effectiveness**—designed to deliver reliable data despite being low-cost.

➢ *Sensors*: Piezoresistive, capacitive, triboelectric, gyroscopes, accelerometers.

**Complexity in data processing**: sensors are characterized by their simplicity in data processing, requiring minimal computational resources and algorithms to interpret and analyze their output

➢ *Sensors*: Accelerometers, gyroscopes, pressure sensors, capacitive sensors, triboelectric sensors.

## 4. Sensors for Non-Specific Parameters in Rehabilitation: A Classification Based on Type, Measured Data, Usability, and Ergonomics

**Figure 5:**
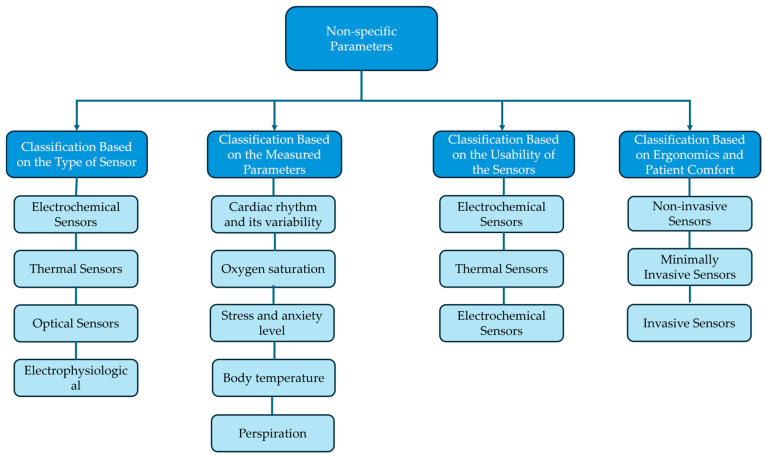
A classification of the non-specific parameters in neuromotor robot-assisted rehabilitation.

Non-specific parameters are defined as physiological and metabolic indicators that do not directly measure motor function but provide essential insights into a patient’s condition, influencing their ability to engage in and respond to therapy. This section provides a structured classification of non-specific parameters and explores their role in adapting rehabilitation strategies to individual patient needs. A schematic representation of the non-specific parameters can be seen in Figure 5.

### 4.1. Classification Based on the Type of Sensor

**Electrophysiological Sensors**

➢ Electrocardiogram (ECG) sensors indicate tolerance to effort and physiological stress [18,72].
➢ Photoplethysmography (PPG) sensors measure blood volume changes in tissues using light, detecting heart rate, oxygen saturation, and vascular health, and they can be used in neurorehabilitation for heart rate variability (HRV) monitoring, biofeedback training, sleep and fatigue tracking, and blood flow assessment [73,74,75].

**Optical Sensors**

➢ Pulse oximeters measure blood oxygen saturation (SpO_2_) and heart rate using light absorption through a fingertip or earlobe [76,77].
➢ NIRS (Near-Infrared Spectroscopy) sensors—measure tissue oxygenation and blood flow by detecting how near-infrared light is absorbed and scattered in biological tissues [18,78].

**Thermal Sensors**

➢ Infrared thermometers: An infrared thermometer measures temperature without contact by detecting infrared radiation emitted by an object or body [79,80].
➢ Skin temperature sensors measure the temperature at the skin’s surface, often using thermistors or infrared technology for health monitoring and thermal regulation [81,82].

**Electrochemical Sensors**: Sweat analysis sensors detect biomarkers like electrolytes, glucose, and cortisol in sweat to monitor hydration, stress levels, and metabolic health in real time [83,84,85].

### 4.2. Classification Based on the Measured Parameters

**Cardiac rhythm and its variability** indicate tolerance to effort and physiological stress, and can be monitored by ECG sensors [18,72].

**Oxygen saturation** is essential for monitoring the effectiveness of exercises in patients with respiratory or cardiovascular conditions. The most commonly used technique for measuring oxygen in the blood and tissues is near-infrared spectroscopy (NIRS) [18,78].

**Stress and anxiety level**—determined through galvanic skin response (GSR) and heart rate variations [18,57,72,76,86,87,88].

**Body temperature**: Besides detecting infections and inflammations inside the body, its temperature can provide information related to the intensity of physical exercises. Given the nature of the treatment, the most suitable sensors for this type of measurement are those integrated into portable devices or those based on detecting the infrared radiation emitted by the skin surface [89].

**Perspiration**: Monitoring sweat during recovery exercises, using sensors integrated into composite materials [86] or patches [87,88], provides valuable insights into a patient’s physical activity, body temperature regulation, hydration status, and psychological condition, as sweating reflects responses to both physical exertion and stress or anxiety.

### 4.3. Classification Based on the Usability of the Sensors

**Wearable Sensors**—compact, body-worn devices that monitor physiological signals such as heart rate, temperature, movement, and biochemical markers for health and performance tracking.

➢ *Sensors*: ECG patches, wrist-worn PPG sensors, smart textiles.

**Stationary Sensors**—devices integrated into rehabilitation equipment for continuous monitoring.

➢ *Sensors*: Force plates, EMG sensors, infrared motion capture systems, fixed PPG sensors, NIRS sensors, thermal cameras, fixed sweat analysis sensors

**Non-contact Sensors** measure physiological parameters without direct skin contact, using technologies like infrared, radar, or optical sensing to track vital signs, movement, and environmental factors.

➢ *Sensors*: Infrared thermometers, camera-based respiration monitors, infrared motion capture systems.

### 4.4. Classification Based on Ergonomics and Patient Comfort

**Non-invasive Sensors**—optical and bioelectrical sensors (e.g., pulse oximeters, ECG patches).

**Minimally Invasive Sensors**—sweat sensors, micro-needle biosensors.

**Invasive Sensors (used in clinical settings**)—implanted metabolic monitoring devices.

*Sensors*: Implantable glucose sensors, intracortical electrodes, microelectrode arrays (MEAs), epidural spinal cord stimulators, intravascular pressure sensors, neurochemical sensors (e.g., for dopamine or glutamate monitoring), myoelectric sensors (implanted EMG electrodes) [90,91,92].

## 5. Classification Based on Comorbidities and Their Impact on Treatment Personalization

In addition to the classification based on specific and non-specific parameters, another important factor in treatment personalization is the presence of comorbidities. These conditions can significantly influence both neuromotor recovery and the patient’s ability to sustain the proposed methods of therapy [93,94,95,96].

**Figure 6:**
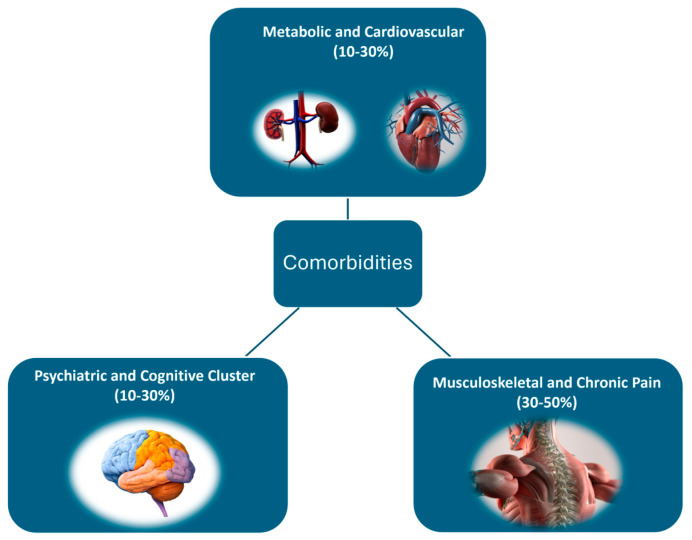
A classification based on associated comorbidities.

In the context of neuromotor robot-assisted rehabilitation, patients frequently present comorbidities that impact the recovery process. Based on a review of relevant studies, three major clusters (Figure 6) of comorbidities can be identified:

**Metabolic and Cardiovascular Cluster (10–30%)**—This group includes patients with hypertension, diabetes mellitus, obesity, and cardiovascular diseases (e.g., atrial fibrillation, previous myocardial infarction). These conditions can interfere with the rehabilitation progress due to cardiovascular limitations and reduced mobility.

**Musculoskeletal and Chronic Pain Cluster (30–50%)**—Patients in this category suffer from arthritis, osteoporosis, chronic lower back pain, and degenerative joint diseases. Chronic pain and joint limitations can significantly affect physical therapy efficiency and increase the risk of accidents.

**Psychiatric and Cognitive Cluster (10–30%)**—This cluster includes individuals with depression, anxiety, mild cognitive impairments, and early-stage dementia. These conditions can negatively influence motivation, concentration, and overall responsiveness to therapy.

These percentage estimations were derived from a synthesis of multiple studies analyzing comorbidity prevalence in neuromotor rehabilitation patients, incorporating data from stroke recovery research, multimorbidity clustering analyses, and reports on musculoskeletal, metabolic, and psychiatric conditions [94,95,97,98,99].

To further understand the impact of these clusters on rehabilitation, it is essential to examine how specific conditions within each category directly affect neuromotor recovery and the technological approaches used for their monitoring.

### 5.1. Metabolic and Cardiovascular Comorbidities

These conditions affect exercise tolerance, muscle function, and vital parameters [94,95].

➢ Diabetes (Type 2 diabetes, blood sugar imbalances) → Peripheral neuropathy, decreased muscle strength, and delayed reaction time.

*Sensors:* EMG, pressure sensors, GSR (galvanic skin response) for autonomic response monitoring, wearable biosensors for glucose monitoring.

➢ Cardiovascular diseases (e.g., hypertension, heart failure) → Reduced exercise tolerance, increased risk of complications.

*Sensors:* ECG, PPG (Photoplethysmography), pulse oximeter for cardiac function monitoring.

### 5.2. Musculoskeletal and Chronic Pain Comorbidities

➢ Neuropathic Pain—Pain resulting from nerve damage due to conditions such as diabetes, spinal cord injury, or multiple sclerosis. Neuropathic pain can alter the way a patient perceives touch, movement, and pressure, making certain movements or exercises intolerable or extremely painful.

*Sensors:* **Accelerometers:** To monitor movement patterns and adjust exercises to minimize strain on the affected nerves.

- **Force Sensors**: To measure how much force is being applied during exercises, ensuring it does not aggravate the pain.

➢ Osteoporosis—A condition characterized by weak, brittle bones, commonly seen in the elderly [93]. Osteoporosis increases the risk of fractures even with minimal pressure or movement, which can make physical rehabilitation difficult.

*Sensors*: **Goniometers**: To track joint range of motion and ensure exercises do not place excessive strain on fragile bones.

- **Accelerometers:** To monitor movement intensity and avoid high-impact actions that could lead to fractures.
- **Force Sensors**: To track the amount of pressure being placed on bones during exercises, that it remains within safe limits.

➢ Muscle Weakness—Weakness in specific muscle groups, often due to neurological conditions like stroke, spinal cord injury, or chronic diseases such as muscular dystrophy. Muscle weakness can severely limit a patient’s ability to perform daily activities and participate in rehabilitation exercises

*Sensors*: **Accelerometers:** To monitor physical activity and detect compensatory movements that the patient may use to avoid using weak muscles.

- **Goniometers:** To track joint movement and range of motion, ensuring that exercises do not strain weakened muscles.
- **Force Sensors**: To measure the applied force during exercises, ensuring that muscle activity is gradual and within the patient’s capacity.

### 5.3. Psychiatric Comorbidities

➢ Depression can arise due to chronic pain, limited mobility, uncertainty about recovery, fatigue, social isolation, medication side effects, and the psychological stress of dealing with long-term illness and rehabilitation. Depression can significantly affect a patient’s motivation to engage in physical activities and adhere to a treatment plan [94].

*Sensors*: **Heart Rate Variability (HRV) Monitor**: Tracks fluctuations in heart rate and autonomic nervous system activity, which can indicate emotional stress or depressive states.

- **Wearable EEG** monitors brainwave activity to detect depressive patterns and guide adjustments in therapy or mental health support.
- **Sleep-Monitoring Devices** help assess the impact of depression on sleep patterns, as poor sleep can worsen depressive symptoms and overall physical function.

➢ Anxiety—anxiety can create heightened physical responses such as muscle tension, increased heart rate, and shortness of breath, which can interfere with therapy.

*Sensors*: **Heart Rate Variability (HRV) Monitor:** This can be used to track the patient’s autonomic responses to anxiety and adjust the therapy accordingly. A drop in HRV may indicate heightened anxiety or stress [94].

- **Wearable EEG**: this is used to monitor brain activity, particularly during moments of stress or anxiety, helping to identify anxiety patterns and optimize the treatment program.
- **Sleep-Monitoring Devices**: Anxiety can severely affect sleep, and these devices can monitor sleep disturbances, which are common in anxious individuals.

➢ Sleep disorders: Due to chronic pain, anxiety, depression, medication side effects, and physical limitations, all of which disrupt normal sleep patterns, sleep disorders can lead to fatigue, reduced recovery time, and difficulty concentrating or performing physical exercises [95].

*Sensors*: **Sleep-Monitoring Devices**: These help track sleep patterns to assess how well the patient is resting and whether sleep disturbances are impacting their recovery. These devices can provide feedback that allows adjustments to the rehabilitation schedule.

**Heart Rate Variability (HRV) Monitor**: This can indicate the impact of poor sleep on the autonomic nervous system, helping identify stress and anxiety levels that may affect the rehabilitation process.

## 6. Real-Time Data Acquisition—Collection Strategies

To better understand the role of the collected parameters, in the context of patient-centered rehabilitation [100,101,102,103,104], an evaluation of real-time acquisition strategies is necessary. These aspects serve as key benchmarks, assisting developers of robotic rehabilitation systems, sensor designers, and medical professionals in delivering personalized treatment solutions.

Processing, storing, and transmitting data from multiple sensors in real-time is a complex and resource-intensive task [105,106]. Each type of sensor generates data with different characteristics, requiring dedicated processing techniques. For example, EEG and EMG signals are particularly susceptible to artifacts caused by muscle activity, motion, and environmental interference, which can obscure meaningful patterns [107]. Removing these artifacts requires the use of adaptive filtering methods, such as the following:

Notch filters—for removing powerline interference (50/60 Hz) [108,109].

High-pass and low-pass filters—for eliminating baseline drift and high-frequency noise [110,111,112].

Independent Component Analysis (ICA)—for separating neural signals from non-neural noise [113,114].

In addition, sensor fusion techniques are increasingly used to enhance accuracy by integrating data from multiple sources. For instance, IMU data can complement EEG recordings by distinguishing intentional movements from involuntary artifacts, improving the interpretation of motor recovery progress [115].

**Figure 7:**
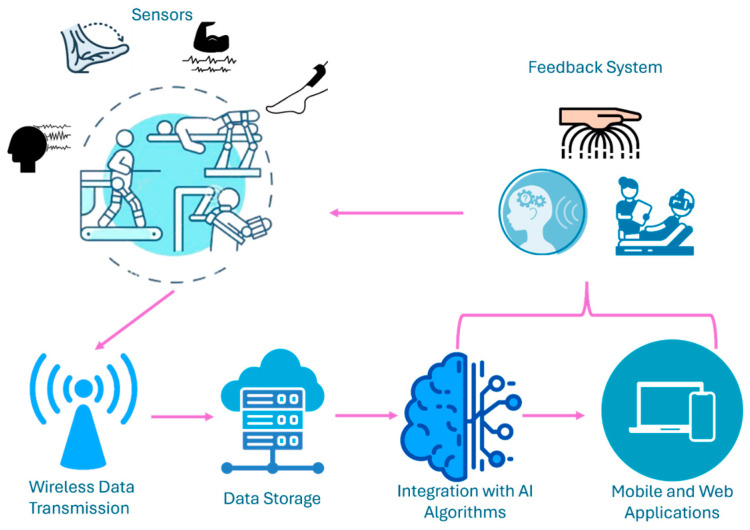
The proposed strategy of data collecting for a patient-centered treatment in neuromotor robot-assisted rehabilitation.

Considering the medical nature of the data of interest, the collection and processing of information regarding neuromotor robot-assisted rehabilitation must consider a series of strategies (Table 2) and aspects, such as the acquisition method, transfer time, data processing, and security. Thus, this subsection presents a series of strategies (schematic representation in Figure 7) that take all these aspects into account and succinctly present the manner in which data collection and processing are carried out for rehabilitation purposes.

The effectiveness of real-time data acquisition is strongly dependent on preprocessing methods, such as noise reduction, adaptive filtering, and feature extraction. In robot-assisted rehabilitation, these techniques allow for cleaner signals and more accurate patient monitoring.

Furthermore, AI-driven analysis models are increasingly being employed for the following:

**Pattern recognition in EMG and EEG data**—to identify movement intention.

**Anomaly detection**—for recognizing irregularities in heart rate, oxygen saturation, or muscle contractions.

**Automated decision-making**—for adjusting therapy intensity based on patient feedback.

**Compression algorithms**—for reducing the size of real-time transmitted data while preserving signal integrity.

Future advancements should focus on optimizing cloud-based storage solutions to accommodate large datasets while ensuring data security, interoperability, and real-time accessibility for clinicians.

## 7. Discussion

### 7.1. Sensor-Driven Personalization of Rehabilitation Strategies

In neuromotor robot-assisted rehabilitation, the effectiveness of a treatment plan is highly dependent on the ability to adapt exercises based on real-time feedback. The classification of sensors into specific and non-specific categories facilitates this adaptability by distinguishing between motion-related metrics and physiological factors influencing recovery. Specific sensors such as IMUs, force sensors, and EMG devices provide critical biomechanical feedback that guides robotic-assisted rehabilitation, while non-specific sensors monitoring physiological parameters (e.g., heart rate variability, oxygen saturation, skin temperature) allow clinicians to make informed decisions regarding therapy intensity and patient readiness. This aligns with the findings of Pan et al. [15], who focused on sensor-based decision-making for safe robot-assisted rehabilitation. However, unlike their approach, which emphasizes safety mechanisms, our classification extends the analysis to both specific and non-specific parameters, ensuring a more comprehensive patient-centered rehabilitation model.

For instance, a patient recovering from a stroke may exhibit varying levels of muscle activation and joint mobility, necessitating continuous adjustment of therapy intensity. EMG sensors can monitor muscle activity to determine whether a patient is engaging in the correct muscle groups, while IMUs can track movement patterns to identify compensatory behaviors. In parallel, non-specific sensors such as ECG or NIRS can provide insights into cardiovascular response, ensuring that the rehabilitation exercises do not exceed the patient’s physiological capacity. Virtual reality (VR) environments can complement this process by providing immersive feedback and interactive exercises that enhance motor recovery and patient engagement. By integrating VR with real-time sensor data, rehabilitation programs can become more adaptable and motivating for patients.

Additionally, in this scenario, an AI-driven rehabilitation system could dynamically adjust resistance levels in robotic-assisted exercises or suggest modifications to therapy based on real-time physiological feedback.

In contrast, a patient with Parkinson’s disease undergoing gait rehabilitation presents unique challenges related to movement fluidity and tremor management. Optical motion tracking combined with IMUs can monitor stride length and step cadence, offering precise metrics to tailor walking exercises. Additionally, EEG sensors may help assess neurophysiological responses to therapy, identifying fluctuations in cognitive-motor control. By integrating these data, therapists can fine-tune interventions, such as adjusting treadmill speed in robotic gait training or implementing cue-based movement strategies to improve walking stability.

The successful implementation of the proposed sensor-based rehabilitation framework relies on interdisciplinary collaboration between medical professionals, biomedical engineers, and AI developers. Clinicians require intuitive interfaces that translate sensor data into actionable insights, facilitating personalized treatment plans. Engineers must optimize sensor design for usability and accuracy, ensuring seamless integration with rehabilitation robotics. Meanwhile, AI developers play a crucial role in refining machine learning algorithms that process sensor-derived data to predict patient progress and adapt therapy in real-time. Establishing a collaborative ecosystem where medical expertise informs AI-driven decision-making will enhance the practical application of this framework, ultimately improving rehabilitation efficiency and accessibility.

### 7.2. Addressing Comorbidities in Sensor-Assisted Rehabilitation

The presence of comorbidities can significantly influence a patient’s ability to participate in rehabilitation. These findings align with those of Kumar et al. [87], who analyzed the impact of comorbidities on recovery outcomes in brain injury rehabilitation. However, unlike their study, which focused on general health factors, our classification provides a structured approach to sensor selection based on specific comorbid conditions such as cardiovascular diseases, metabolic disorders, and psychiatric conditions, thus facilitating personalized rehabilitation plans. For example, patients with diabetes often experience peripheral neuropathy, reducing proprioceptive feedback and increasing the risk of balance issues. Pressure sensors embedded in rehabilitation footwear can help assess plantar pressure distribution, ensuring that exercises do not exacerbate foot ulcers or contribute to further sensory loss. Additionally, real-time glucose monitoring via electrochemical sensors can inform clinicians of any metabolic fluctuations that may impact therapy endurance.

Similarly, in patients with cardiovascular disease, excessive exertion during rehabilitation can pose a risk. Monitoring heart rate variability and oxygen saturation in real time using ECG and pulse oximeters allows for the modification of therapy intensity to prevent overexertion. For example, in a post-stroke patient with a history of hypertension, an AI-driven system could reduce the intensity of robotic-assisted arm exercises when a sudden increase in heart rate variability is detected, preventing potential cardiovascular strain.

Psychiatric conditions such as depression and anxiety also impact rehabilitation adherence. Patients with high anxiety levels may exhibit increased muscle tension, potentially leading to inefficient movement patterns and discomfort during therapy. Wearable EEG sensors or HRV monitors can help assess stress responses, allowing therapists to integrate relaxation techniques or adjust session pacing to enhance patient comfort. Furthermore, sleep-monitoring sensors can identify disturbances that may contribute to fatigue and reduced motivation, enabling the scheduling of therapy sessions at optimal times for patient engagement.

While the proposed classification framework provides a structured approach to sensor-based neuromotor rehabilitation, further research is needed to validate its effectiveness in real-world settings. Currently, no large-scale experimental validation has been conducted, but future studies will focus on assessing the practical applicability of this framework in clinical environments. Planned research efforts include pilot studies and clinical trials aimed at evaluating how integrating both specified and non-specified sensor parameters impacts patient recovery. Additionally, collaborations with rehabilitation centers are being explored to test the framework’s feasibility and adaptability in therapy sessions. These steps will provide essential insights into its clinical relevance and long-term impact on patient outcomes.

### 7.3. Enhancing Treatment Outcomes Through AI-Driven Data Processing

The integration of AI-driven data processing further strengthens sensor-based rehabilitation by automating the interpretation of complex data patterns. By combining sensor inputs with machine learning models, rehabilitation systems can predict a patient’s progress and suggest personalized modifications.

For example, in robot-assisted therapy for spinal cord injury (SCI), sensors tracking muscle activation and joint movement can identify small improvements in voluntary control that may not be immediately apparent to therapists. AI algorithms can detect these subtle trends and recommend modifications to therapy parameters, such as increasing movement resistance or adjusting neuromuscular stimulation patterns to maximize neuroplasticity.

In telerehabilitation, AI can influence wearable sensor data to provide real-time feedback to remote patients. A system using motion sensors and force sensors can assess home-based exercise execution, providing corrective feedback via a mobile application. Additionally, by analyzing past sensor data, AI can predict potential therapy plateaus and suggest alternative exercises to maintain patient progress.

AI-driven rehabilitation has shown significant potential in both sports medicine and clinical rehabilitation. Studies highlight its role in injury prevention, training optimization, and adaptive recovery strategies [138]. Additionally, a systematic review emphasizes that AI-supported rehabilitation technologies, such as robotic devices, gaming systems, and wearables, are promising but still require rigorous clinical validation. These findings support the need for further integration of AI into sensor-based neuromotor rehabilitation frameworks [127].

### 7.4. Future Considerations in Sensor-Assisted Neuromotor Robot-Assisted Rehabilitation

Despite the advantages of sensor-assisted rehabilitation, challenges remain in ensuring widespread clinical adoption and optimizing data-processing strategies. One critical aspect is interoperability—ensuring that data from different sensors (e.g., IMUs, EEG, force sensors) can be integrated into a unified rehabilitation platform. Standardization of sensor data formats and communication protocols is essential for developing holistic rehabilitation frameworks. Additionally, integrating virtual reality (VR) into sensor-assisted rehabilitation programs presents an opportunity to enhance therapy adherence and effectiveness by creating engaging rehabilitation scenarios tailored to patient needs. Furthermore, imaging studies, such as functional MRI (fMRI), play a critical role in shaping rehabilitation strategies. Although these studies are typically conducted over short periods (1–2 h), they provide valuable insights into brain activity and neural plasticity. This information helps in designing personalized therapy plans and long-term recovery approaches. Moreover, incorporating other imaging techniques like PET scans can further enhance our understanding of neurophysiological changes during rehabilitation, supporting the development of more tailored and effective therapeutic interventions.

Another consideration is long-term patient adherence. While wearable sensors and robotic systems provide valuable insights, patient comfort and ease of use are crucial for sustained engagement. Lightweight, non-invasive sensor designs, along with user-friendly interfaces, will be essential in encouraging consistent use of sensor-based rehabilitation solutions.

Furthermore, ethical considerations regarding data privacy must be addressed. The continuous collection of physiological and biomechanical data raises concerns regarding data security, necessitating robust encryption and anonymization protocols. Transparent data-sharing policies should be established to ensure that patient information is protected while still enabling collaborative advancements in rehabilitation technology.

## 8. Conclusions

The scientific literature has focused on the role of sensors in neuromotor robot-assisted rehabilitation, analyzing their ability to monitor specific parameters such as motion, force, and muscle activity, as well as non-specific parameters like oxygen saturation and heart rate. However, existing studies have not provided a unified classification framework that integrates these sensor types into a patient-centered rehabilitation model. Furthermore, while various approaches have explored the use of AI and real-time feedback in robotic-assisted therapy, they have not established a structured methodology for selecting and integrating sensors in a way that accounts for individual patient conditions.

This study addresses these gaps by proposing a novel classification framework that systematically categorizes sensors based on their type, measured data, usability, and impact on rehabilitation. It introduced a personalized approach that considers comorbidities—such as cardiovascular, metabolic, and psychiatric conditions—to optimize sensor selection and therapy adaptation. Additionally, we outline an AI-driven data acquisition and processing strategy that enables real-time adjustments in robotic-assisted rehabilitation, ensuring a more dynamic and patient-specific treatment process.

## Figures and Tables

**Table 1:** Targeted parameters in robot assisted rehabilitation.

Robot	Upper Limb	Lower Limb
Joint torque [62,63,64]	EEG—electrical activity of the brain [59,60,61]	EEG—electrical activity of the brain [59]
Joint angle [26,33]	EMG—muscle activity [55,56]	EMG—muscle activity [57]
Joint stiffness [65]	Range of motion [26]	Range of motion [27,66]
Pressure distribution [67,68]	Maximum isometric force [42]	Ground interaction force [41]
Force feedback [66]	Muscle contraction length [53]	Muscle contraction length [46]
	Muscle activation [58]	Muscle activation [57]
	Angle for specific motions (abduction/adduction; rotation; pronation/supination; flexion extension) [26]	Inclination angle [28]
	Angular velocity [26]	Angular velocity [26]
	Linear acceleration [19]	Linear acceleration [19]
	Angular acceleration [29]	Angular acceleration [66]

**Table 2:** The proposed strategy for robot-assisted rehabilitation.

Strategy	Key Steps and Considerations
Sensors	**Sensor Selection:** Choosing sensors based on the targeted signals [116]. **Placement Optimization**: Determining the most suitable sensor placement areas to ensure comfort for patients, easy accessibility for replacement, and maximum efficiency in data collection. (e.g., a certain muscle for EMG) [117,118]. **Calibration and Synchronization**: Implement calibration protocols and synchronization methods [119].
Filtering and Artifact Removal	**1.** **Identification of motion artifacts,** especially in EEG and EMG caused by involuntary movements, electrode displacement, and external vibrations [120,121]. **2.** **Separation of physiological noise,** such as muscle contractions, and cardiac activity from neural signals [120]. **3.** **Real-time monitoring and adaptive artifact correction,** enabling dynamic adjustments during data acquisition, [120,122]. **4.** **Development of patient-specific filtering models** that adjust artifact detection based on individual physiological characteristics.
Wireless Data Transmission	**Technology Selection**: Choosing a data transmission method that ensures real-time transmission, minimal loss, and compatibility with other communication protocols in the intended environments [123,124]. **Reducing delay** times in case they exist. **Signal Reliability: Reducing interference with other signals.**
Data Storage	**Cloud Provider Selection**: Selecting methods for massive data storage, with the possibility of access by multiple users (cloud storage) [69,124,125]. **Data Architecture**: Creating an architecture that facilitates data organization. **Security Measures**: Implementing data protection methods such as data encryption, authentication and authorization, regular Backups, Access Monitoring, Data Anonymization and Pseudonymization [126] **Backup**: Developing methods for data backup in case of accidental loss. Developing methods for data backup in case of accidental loss.
Integration with AI Algorithms	**Data Selection**: Selecting data that can highlight certain patterns, correlations, or behaviors considered pathological [72,127,128]. **Data processing**: Data cleaning in order to keep just the relevant data, **Algorithm Selection**: Selecting algorithms that ensure both adequate results and optimized learning. **Real-Time Processing**: Implement real-time data processing frameworks [129]. **Feedback Loop**: Develop systems for real-time feedback and adaptive rehabilitation protocols.
Mobile and Web Applications	**User Interface Design:** Developing user-friendly interfaces for both patients and physicians [130]. **Real-Time Data Visualization**: Implementing real-time data visualization methods. **Remote Monitoring Capabilities**: Development of technologies enabling telerehabilitation [131,132].
Feedback Systems	**Haptic Feedback Devices**: Develop or integrate devices providing tactile feedback [133,134]. **Visual and Auditory Cues**: Design systems for visual and auditory feedback to guide patient movements including AR and VR devices [135,136,137]. **Personalized Feedback**: Usage AI algorithms for personalized feedback based on patient progress and predictions [72,78].

## Data Availability

The original contributions presented in the study are included in the article; further inquiries can be directed to the corresponding author.
